# Guanylate Binding Protein 1 Inhibits Osteogenic Differentiation of Human Mesenchymal Stromal Cells Derived from Bone Marrow

**DOI:** 10.1038/s41598-018-19401-2

**Published:** 2018-01-18

**Authors:** Shi Bai, Zhixiang Mu, Yuanding Huang, Ping Ji

**Affiliations:** 1 Stomatological hospital of Chongqing Medical University, Chongqing, China; 2 Chongqing Key Laboratory of Oral Diseases and Biomedical Sciences, Chongqing, China; 3 Chongqing Municipal Key Laboratory of Oral Biomedical Engineering of Higher Education, Chongqing, China

## Abstract

Guanylate Binding Proteins (GBPs) are a group of cytokine-inducible large guanosine triphosphatase. Previous studies have shown high expression of *GBP1* in circulating monocytes of premenopausal subjects was correlated to extremely low peak bone mass, which is considered as an important determinant of osteoporosis. However, whether GBPs play a role in regulation of osteogenesis of mesenchymal stromal cells (MSCs) remains largely unknown. In the present study, we found that mRNA expression of *GBP1* was highest among all the *GBP*s, and it was dramatically downregulated during osteogenic differentiation of human MSCs derived from bone marrow (hBM-MSCs). While siRNA-mediated knockdown of *GBP1* promoted osteogenesis, overexpression of *GBP1* suppressed osteogenesis of hBM-MSCs. Furthermore, we found GBP1 is required for expression of *indoleamine 2,3 dioxygenase* (*IDO*), *Interleukin 6* (IL-6) and *IL-8* induced by treatment with Interferon-γ (IFN-γ). Depletion of *GBP1* rescued the inhibited osteogenesis induced by IFN-γ treatment, at least in part. Collectively, our findings indicate GBP1 inhibits osteogenic differentiation of MSCs, and inhibition of GBP1 expression may prevent development of osteoporosis and facilitate MSC-based bone regeneration.

## Introduction

Osteoporosis is a skeletal disorder affecting 9 million people worldwide, which is characterized by compromised bone strength with an increase in risk of fracture^1,2^. The skeleton is a dynamic organ where bone mass is controlled by a delicate balance between bone resorption by osteoclasts and bone formation by osteoblasts^3^. Mesenchymal stromal cells (MSCs) are a heterogeneous population of multipotent cells that can give rise to osteoblasts, chondrocytes and adipocytes^4–6^. As the progenitors of osteoblasts, mesenchymal stromal cells (MSCs), the osteoblastic differentiation of MSCs are critical for the maintenance of bone mass and stem cell-based bone repair. Several factors, such as parathyroid hormone (PTH) and bone morphogenetic proteins (BMPs), have been used for osteoporosis treatment and bone regeneration, respectively^7,8^. However, the molecular mechanisms governing osteoblastic differentiation of MSCs is still not fully understood. In addition, a lot of effort has been made to identify risk genes for susceptibility to osteoporosis that may facilitate prevention of osteoporosis^9,10^. For instance, human guanylate binding protein 1 (hGBP1) was found to be up-regulated in circulating monocytes of premenopausal subjects with extremely low peak bone mass (PBM), which is considered as an important determinant of osteoporosis^11,12^.

The GBPs are a subfamily of cytokine-induced dynamin superfamily of large guanosine triphosphatase (GTPases)^13–15^. GBPs are capable of binding to agarose-immobilized guanosine triphosphate (GTP), guanosine monophosphate (GMP), and guanosine diphosphate (GMP), and hydrolyzing GTP to both GDP and GMP. To date, 7 h*GBP*s (h*GBP*-1 through -7) have been designated within a cluster on chromosome 1in human genome, while 11 m*GBP*s have been designated in 2 clusters on chromosome 3 and 5 in murine genome, respectively^16^. hGBP-1 through -5 and all the mGBPs can be induced by interferon-gamma(IFN-γ). Thus, GBPs have been extensively used as markers of interferon responsiveness. Furthermore, accumulating evidence has revealed the critical function of the GBPs in various processes^17^. For instance, hGBP1 has been shown to mediate the antibacterial and antiviral activities of IFN-γ. m*Gbp2*, the putative murine homologue of hGBP1, is required for lysis of pathogen-containing vacuoles in bone marrow-derived macrophages (BMMs). mGBP2−/− BMMs exhibited reduced levels of cell death, cytokine secretion and caspase release^18^. In addition, *Ostler et al*. reported that hGBP1 co-localized with actin and regulated remodeling of the fibrous actin structure in IFN-γ-treated Hela cells^19^.

In regard to MSCs, previous study has shown that hGBP1 can be induced by IFN-γ, and play an important role in the immunity of hMSCs against *Toxoplasma gondii*^20^. IFN-γ was also found to inhibit osteogenic differentiation of MSCs^21^. Furthermore, a recent study has revealed that m*Gbp2* was downregulated during osteogenic differentiation of mouse MC3T3 cells, an osteoblast precursor cell line^22^. However, the role of GBPs in osteogenic differentiation of MSCs remains largely unknown. In the present study, we evaluated the expression levels of hGBPs during osteogenic differentiation of MSCs derived from human bone marrow (hBM-MSCs), and investigated the changes in osteogenic differentiation potential of BMSCs in response to knockdown and overexpression of hGBP1, respectively. Finally, we found hGBP1 was induced by IFN-γ treatment, and was required for the upregulation of the target genes induced by IFN-γ in hBM-MSCs.

## Methods and Materials

### Cell Culture

Primary hBM-MSCs were purchased from Rooster Bio (Frederick, MD, USA). The cells were maintained in Dulbecco’s Modified Eagle Medium (DMEM), supplemented with 15% heat-inactivated FBS and 100 U/ml of K-Penicillin G and100 mg/ml of Streptomycin sulfate at 37°C in a humidified atmosphere of 5% CO_2_ (all from Invitrogen, Carlsbad, CA, USA). The cells used in this study were within passages 4–10. 10 ng/ml recombinant human IFN-γ (R&D systems, Minneapolis, MN, USA) was used to treat hBM-MSCs as indicated. To induce osteogenic differentiation of hBM-MSCs, osteogenic stimulus (OS) containing 100 μM ascorbic acid, 10 mM β-glycerophosphate, and 10 nM dexamethasone (all from Sigma-Aldrich, St. Louis, MO, USA) was added into the medium.

### Transfection

All siRNAs were purchased from Invitrogen, including a scrambled-siRNA (siScr) as a control, and siRNA targeting hGBP1 (siGBP1). Transfection was performed using Lipofectamine RNAiMAX reagent (Invitrogen) according to the manufacturer’s instructions. For overexpression of GBP1, retroviruses expressing human GBP1 gene were purchased from Fulengen Inc. (Guangzhou, China). hBM-MSCs were infected with in the presence of polybrene (Sigma) for 24 hours. hBM-MSCs transfected with empty vector were used as control.72 hours post-transfection, 500 µg/ml G418 was added into the medium for 3 days, and the selected cells were expanded in growth medium.

### Characterization of Osteoblastic Phenotypes

Alkaline phosphatase (ALP) staining and Alizarin Red S (ARS) staining were performed after several days of osteogenic induction as previously described^23,24^. For the quantitative measurement of ALP activity, 10 μL cell protein extraction was incubated with 50 μL ALP stabilizing buffer (Sigma) and 50 μL ALP yellow (pNPP) liquid substrate (Sigma) for 20 min at 37 °C. The absorbance was then measured at 405 nm.

### RNA Extraction and Quantitative Reverse Transcription-PCR (RT-qPCR)

Total RNA was isolated using the TRIzol reagent (Invitrogen) according to manufacturer’s instructions. Then, complementary DNA was synthesized with 1 ug aliquots of total RNA using PrimeScript RT Reagent Kit (TAKARA, Otsu, Shiga, Japan). QPCR was performed using SYBR Premix Ex Taq kit (TAKARA).The primer sequences used for this analysis were as following: 5′-GGAGCGAGATCCCTCCAAAAT-3′ (forward) and 5′ -GGCTGTTGTCATACTTCTCATGG-3′ (reverse) for *GAPDH*; 5′-GAAGTGCTAGAAGCCAGTGC-3′ (forward) and 5′-CCACCACCATAGGCTGTGTA-3′ (reverse) for *GBP1*; 5′-CTATCTGCAATTACGCAGCCT -3′ (forward) and 5′ -TGTTCTGGCTTCTTGGGATGA-3′ (reverse) for *GBP2*; 5′ - ATTCCCTGAAGCTAACGCAAG -3′ (forward) and 5′-GGGCAGATCGAAGACAAAACATT-3′ (reverse) for *GBP3*; 5′-ATGGGTGAGAGAACTCTTCACG-3′ (forward) and 5′- TGCGGTATAGCCCTACAATGG -3′ (reverse) for *GBP4*; 5′-CCATGTGCCTCATCGAGAACT-3′ (forward) and 5′-ACAGGTTGCGTAATGGCAGAC-3′ (reverse) for *GBP5*; 5′-AACCATCTGGCAGGACAGAAT-3′ (forward) and 5′-TCACCCTTTTCCACATCGCC -3′ (reverse) for *GBP6*; 5′-GTGGAGCGACTCCTTGTCTG-3′ (forward) and 5′-GTGGGGAATCTCACTTGCTGG -3′ (reverse) for *GBP7*; 5′ - GAGGGCCAAGACGAAGACATC-3′ (forward) and 5′ - CAGATCACGTCATCGCACAAC -3′ (reverse) for *COL1A1*; 5′ - CACTGGAGCCAATGCAGAAGA-3′ (forward) and 5′-TGGTGGGGTTGTAGGTTCAAA-3′ (reverse) for *IBSP*; 5′-GCCGCTGTAACCTCTTCGG -3′ (forward) and 5′-GTCTTCGGCCAATCTGGCTTT -3′ (reverse) for *SPP1*; 5′-ACTCACCTCTTCAGAACGAATTG -3′ (forward) and 5′-CCATCTTTGGAAGGTTCAGGTTG -3′ (reverse) for *IL6*; 5′- CTTTCAGAGACAGCAGAGCAC-3′ (forward) and 5′-ACTGTGAGGTAAGATGGTGGC-3′ (reverse) for *IL8*; 5′-GCCAGCTTCGAGAAAGAGTTG-3′ (forward) and 5′-ATCCCAGAACTAGACGTGCA -3′ (reverse) for *IDO*.

### Western Blot

Western blot was performed as previous described^25^. Briefly, the cells were lysed for 30 min in RIPA buffer (Santa Cruz Biotechnology, Paso Robles, CA, USA), and centrifuged at 18,000 g for 15 min at 4 °C. 30 μg aliquots of the lysates were separated on a 10% sodiumdodecyl sulfate-polyacrylamidegel. The resolved proteins were then transferred onto nitrocellulose membrane (Bio-Rad, Hercules, California, USA). The membrane was subsequently incubated with primary antibodies followed by a horseradish peroxidase (HRP)-conjugated secondary antibody (Boster, Wuhan, China). Protein bands were detected using an enhanced chemiluminescence western blotting detection kit (Thermo, Canoga Park, CA, USA). Antibodies for western blot were purchased from the following suppliers: rabbit polyclonal anti-human GBP1 antibody (Invitrogen), rabbit polyclonal anti-human IDO1 antibody (Invitrogen).

### Statistical Analysis

Data was shown as mean ± SD from three independent experiments. Statistical analysis was performed using Graph Pad Prism 6 software. Student’s t-test and one-way analysis of variance (ANOVA) were performed for single comparisons and multiple comparisons, respectively. The statistical significance of differences among IFN-γ treatments were assessed using two-way ANOVAs with Holm-Sidak post-hoc test. *p < 0.05; **p < 0.01; ***p < 0.001.

## Results

### GBP1 is downregulated during osteogenic differentiation of hBM-MSCs

**Figure 1:**
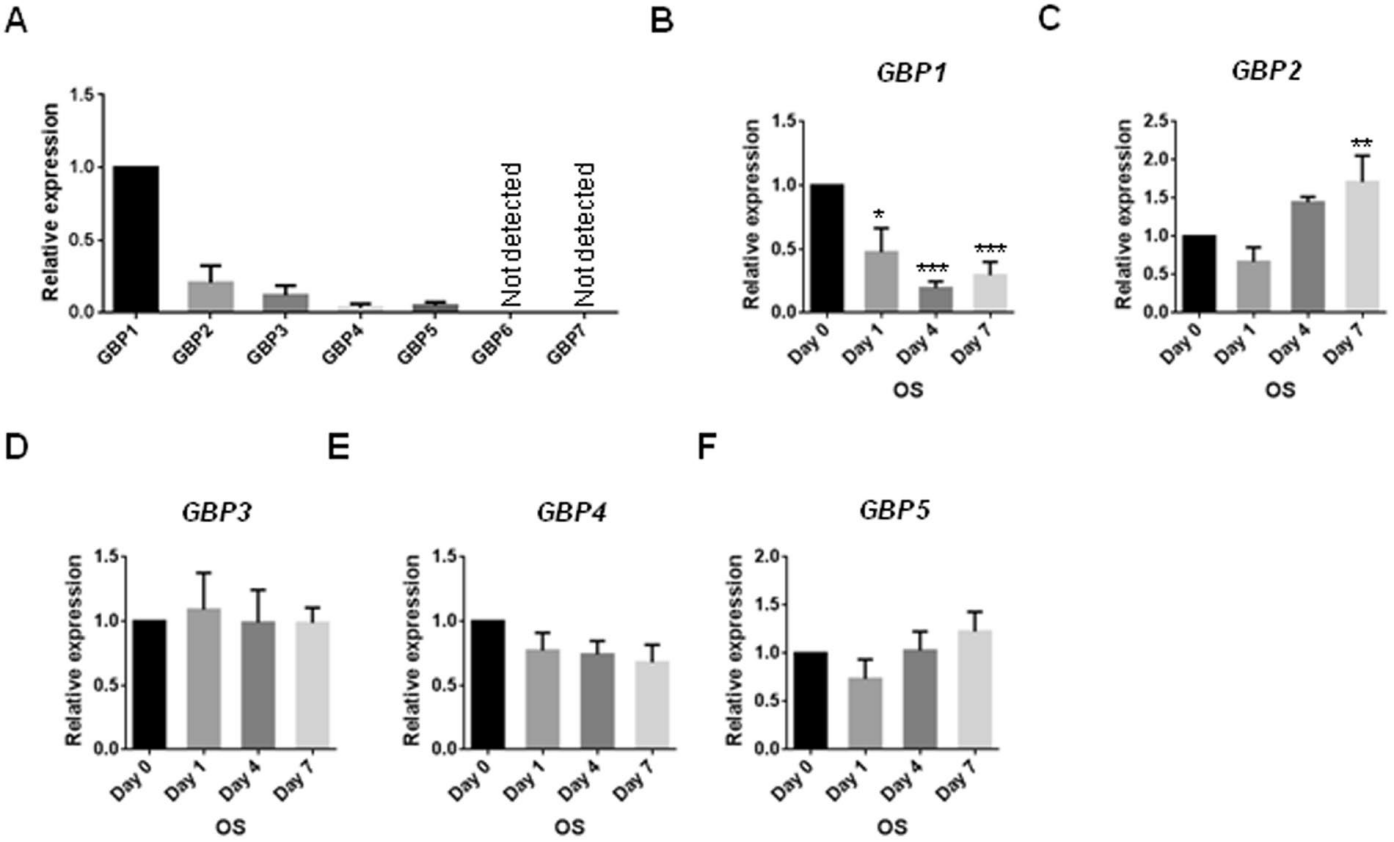
Expression profiling of *GBP*s in hBM-MSCs and during osteogenic differentiation of hBM-MSCs. (**A**) The mRNA expression levels of *GBP*-1through -7 in hBM-MSCs. (**B**–**F**) The mRNA expression levels of *GBP*1 (**B**), *GBP*2 (**C**), *GBP*3 (**D**), *GBP*4 (**E**), *GBP*5 (**F**) in response to osteogenic stimulus (OS) in hBM-MSCs at 0, 1, 4, 7 days. Asterisks indicate a significant difference compared to the baseline. *p < 0.05; **p < 0.01; ***p < 0.001.

To investigate the role of GBPs in regulation of osteogenic differentiation of MSCs, we first screened the expression profiles of h*GBP*s in hBM-MSCs by RT-qPCR. As shown in Fig. 1A, *GBP1* was of highest expression level in all the 7 *GBP*s, and the expression of *GBP* -4 to -7 was also detected. However, the expression of *GBP6* and *GBP7* was not detected by RT-qPCR (Fig. 1A). We next examined the expression of *GBP1* to *GBP5* during the osteogenic differentiation of hBM-MSCs. Notably, expression of *GBP1* was dramatically downregulated upon osteogenic stimulus (OS) in hBM-MSCs (Fig. 1B). While *GBP2* expression was upregulated after 7 days of osteogenic differentiation, expression of *GBP3*, *GBP4*, and *GBP5* seemed to be unchanged during osteogenic differentiation of hBM-MSCs (Fig. 1C to F).

### Depletion of *GBP1* enhanced osteogenic differentiation of hBM-MSCs

**Figure 2:**
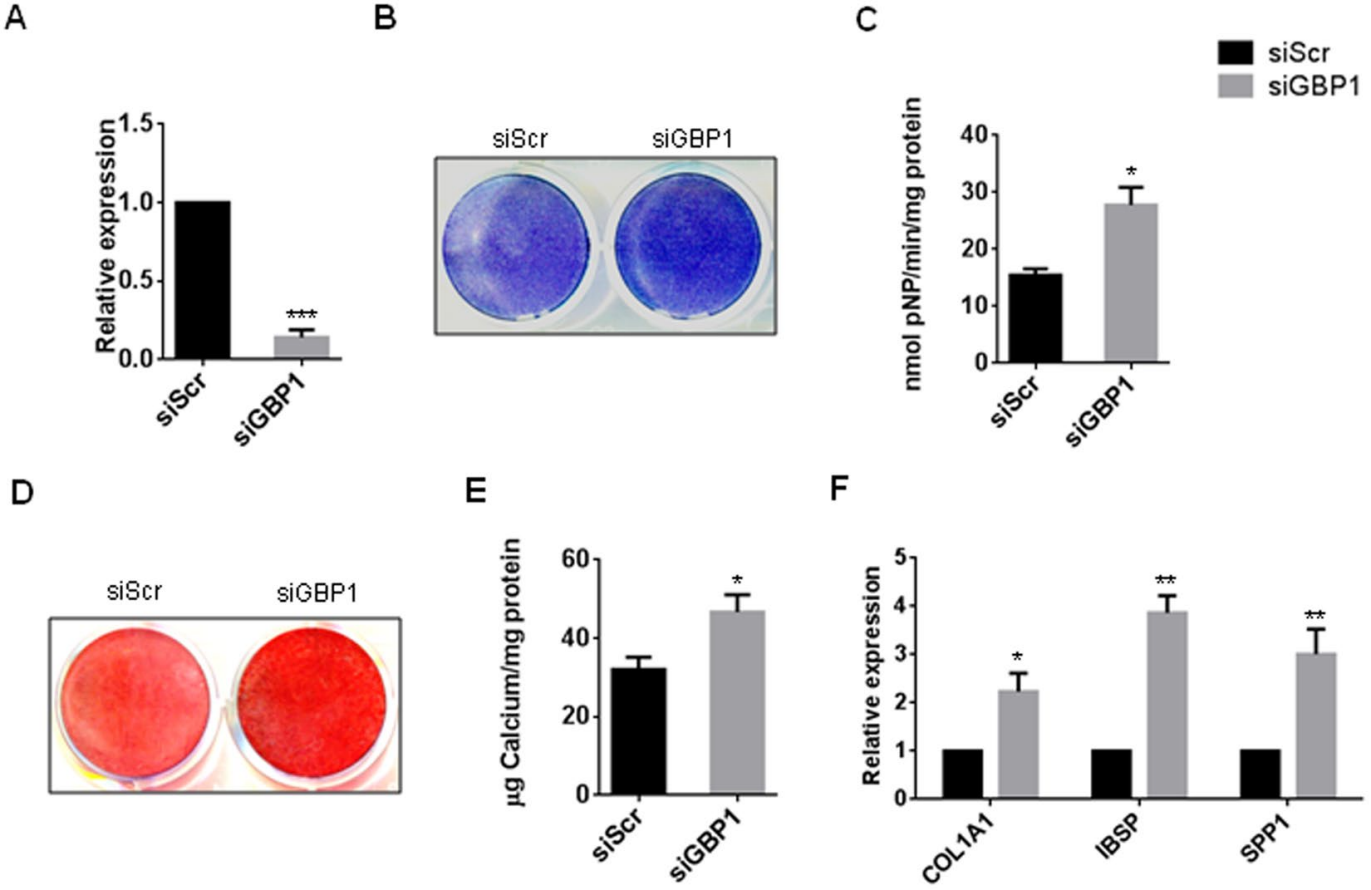
siRNA-mediated depletion of Cdo1 enhances osteogenic differentiation of hBM-MSCs. (**A**) The knockdown efficiency of siRNA targeting *GBP1* compared to scrambled siRNA (siScr) was confirmed by RT-PCR. (**B**) Knockdown of *GBP1* enhanced the ALP staining after 7 days of osteogenic induction in hBM-MSCs. (**C**) Knockdown of *GBP1* enhanced the ALP activity after 7 days of osteogenic induction as determined by quantitative ALP activity assay. (**D**) Knockdown of *GBP1* enhanced mineralization of extracellularmatrix (ECM) after 3 weeks of osteogenic induction. (**E**) Quantification of Alizarin Red S (ARS) staining in D. (**F**) Knockdown of *GBP1* promoted expression levels of *COL1A1* (Collagen, type I, alpha 1), *IBSP* (Integrin binding sialoprotein), and *SPP1* (secreted phosphoprotein 1), as determined by RT-PCR. *p < 0.05; **p < 0.01; ***p < 0.001.

To investigate whether GBP1 inhibits osteogenic differentiation of MSCs, we used specific siRNA to knockdown the expression of GBP1 in hBM-MSCs, and the knockdown efficiency was assessed by RT-PCR and western blot (Fig. 2A). Interestingly, we found that depletion of *GBP1* significantly enhanced ALP activity in hBM-MSCs after 7 days of osteogenic induction (Fig. 2B,C). We further assessed the extracellular matrix (ECM) mineralization by ARS staining after 3 weeks of osteogenic induction. As shown in Fig. 2D and E, the ECM mineralization was also enhanced by depletion of *GBP1*. Furthermore, the expression of osteogenesis markers, such as *COL1A1* (Collagen, type I, alpha 1), *IBSP* (Integrin binding sialoprotein), and *SPP1* (secreted phosphoprotein 1), was elevated by depletion of *GBP1* (Fig. 2F).

### Overexpression of *GBP1* inhibited osteogenic differentiation of hBM-MSCs

**Figure 3:**
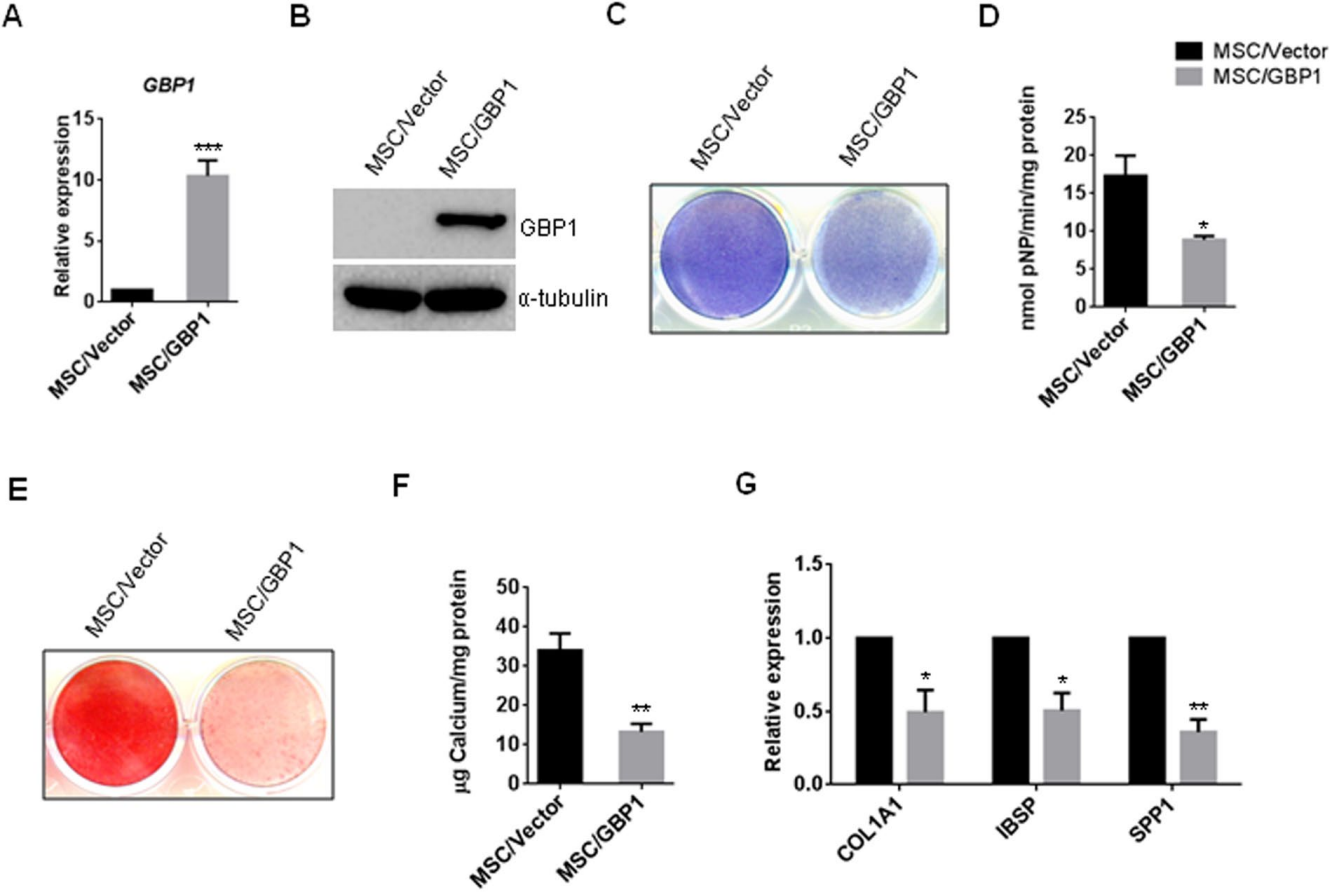
Ectopic overexpression of *GBP1* suppresses osteogenic differentiation of hBM-MSCs. (**A**,**B**) Overexpression of *GBP1* in hBM-MSCs was confirmed by RT-PCR (**A**) and western blot (**B**). Original images of western blots were presented in Supplementary Figure S1. (**C**) Overexpression of *GBP1* inhibited the ALP staining after 7 days of osteogenic induction. (**D**) Overexpression of *GBP1* impaired the ALP activity after 7 days of osteogenic induction. (**E**) Overexpression of *GBP1* reduced mineralization post-3 weeks of osteogenic induction. (**F**) Quantification of ARS staining in (**E**). (**G**) Overexpression of Cdo1 inhibited mRNA expression levels of *COL1A1*, *IBSP*, and *SPP1*. *p < 0.05; **p < 0.01; ***p < 0.001.

Next, hBM-MSCs were stably transduced with retroviruses expressing h*GBP1*, and the overexpression efficiency was assessed by RT-PCR and western blot (Fig. 3A,B). After osteogenic induction, we found overexpression of *GBP1* inhibited ALP activity, as determined by ALP staining and quantitative ALP activity assay (Fig. 3C,D). The ECM mineralization was also significantly reduced by overexpression of *GBP1* in hBM-MSCs (Fig. 3E,F). Consistently, the expression of *COL1A1*, *IBSP*, and *SPP1* are also suppressed in hBM-MSCs after 7 days of osteogenic induction (Fig. 3G). Taken together, these findings demonstrated that GBP1 suppressed osteogenic differentiation of hBM-MSCs.

### GBP1 is required for IFN-γ-induced processing of IDO, IL-6 and IL-8

**Figure 4:**
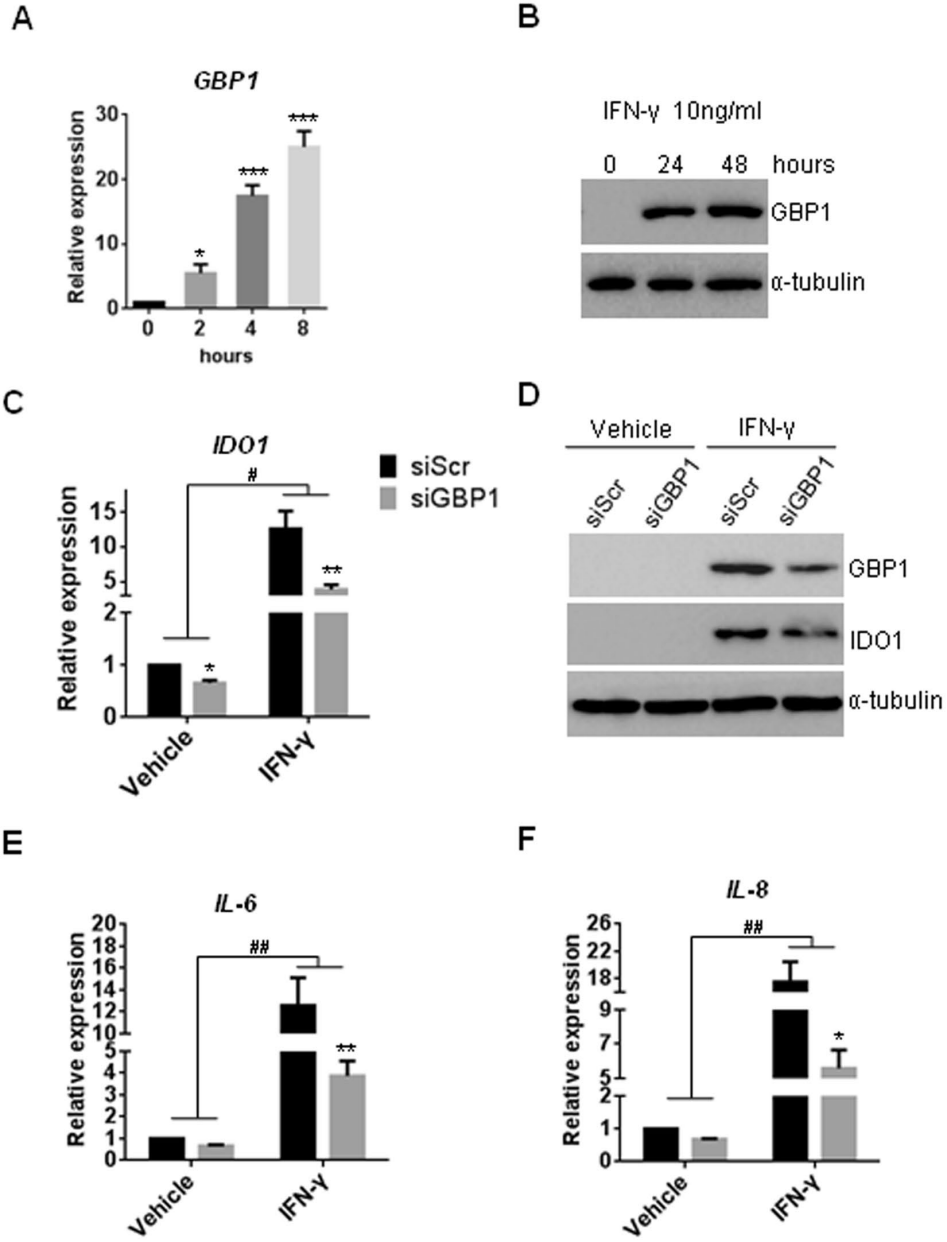
GBP1 is required for IFN-γ-induced processing of IDO, IL-6 and IL-8. (**A**,**B**) Expression of GBP1 was dramatically upregulated by treatment with IFN-γ at 10 ng/ml, as determined by RT-PCR (**A**) and western blot (**B**). Original images of western blots were presented in Supplementary Figure S2A. (**C**) Knockdown of *GBP1* impaired the mRNA expression of indoleamine 2,3 dioxygenase (IDO) induced by treatment with IFN-γ (#p < 0.05 by two-way ANOVA with Holm-Sidak post-hoc test). (**D**) Knockdown of *GBP1* impaired the protein expression of indoleamine 2,3 dioxygenase (IDO) induced by treatment with IFN-γ. Original images of western blots were presented in Supplementary Figure S2B. (**E**, **F**) Knockdown of *GBP1* impaired the mRNA expression of Interleukin 6 (*IL-6*) (E) and *IL-8* (F) induced by IFN-γ treatment (^##^p < 0.01 by two-way ANOVA with Holm-Sidak post-hoc test). *p < 0.05; **p < 0.01; ***p < 0.001.

**Figure 5:**
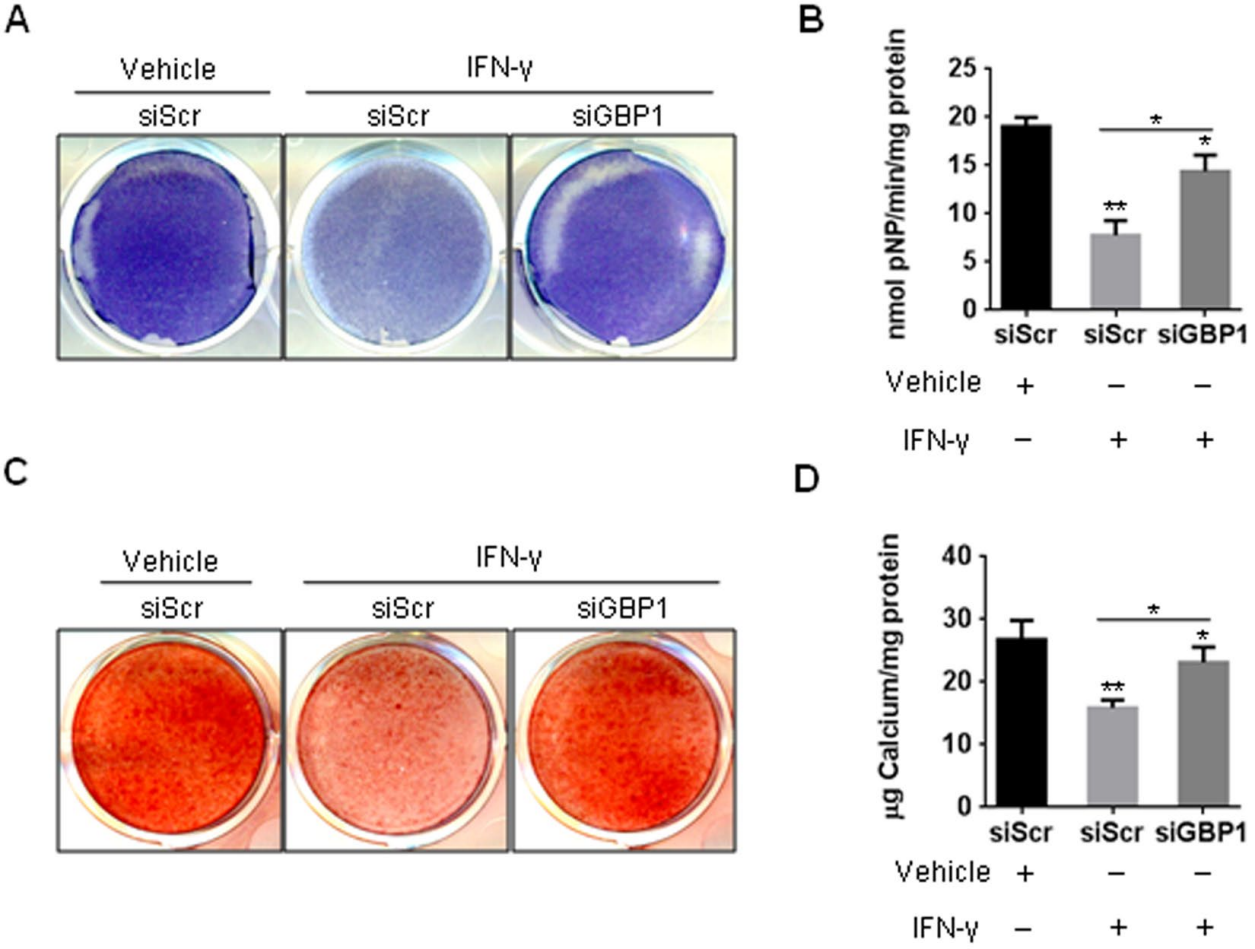
Depletion of *GBP1* partially rescued the inhibited osteogenesis by IFN-γ treatment in hBM-MSCs. (**A**,**B**) Knockdown of GBP1 partially restored the inhibited ALP activity by IFN-γ treatment, as determined by ALP staining (**A**) and quantitative ALP activity assay (**B**). (**C**) Knockdown of GBP1 partially rescued the reduced ECM mineralization by IFN-γ treatment. (**D**) Quantification of ARS staining in (**D**). *p < 0.05; **p < 0.01; ***p < 0.001.

Previous study has reported that IFN-γ treatment induced expression of GBPs, and suppressed osteogenic differentiation of human MSCs from bone marrow via activation of indoleamine 2,3 dioxygenase (IDO)^20,21^. Consistently, we found GBP1 was upregulated in response to treatment with IFN-γ at 10 ng/ml (Fig. 4A,B). As revealed by RT-PCR and western blot assay, we found knockdown of *GBP1* significantly inhibited expression of IDO in the absence or presence of IFN-γ (Fig. 5C,D). Furthermore, knockdown of *GBP1* also suppressed expression of Interleukin 6 (*IL-6*) and *IL-8* induced by IFN-γ treatment (Fig. 5E,F), indicating GBP1 may play an important role in IFN-γ signaling, and GBP1 may inhibit osteogenesis of MSCs by regulating IFN-γ signaling.

### Depletion of *GBP1* partially rescued the inhibited osteogenesis by IFN-γ treatment in hBM-MSCs

To investigate whether the induction of GBP1 is required for the inhibitory effect of IFN-γ on osteogenesis, hBM-MSCs were transduced with siRNA targeting GBP1 for 24 h before treatment with IFN-γ. Interestingly, we found knockdown of GBP1 rescued the inhibited ALP activity of hBM-MSCs by IFN-γ treatment, at least in part (Fig. 4A,B). In addition, knockdown of GBP1 also partially restored the reduced mineralization of hBM-MSCs after 3 weeks of osteogenic induction (Fig. 4C,D). Collectively, our findings suggest IFN-γ-inducible GBP1 inhibits osteogenic differentiation of human MSCs.

## Discussion

Understanding of molecular mechanisms that regulate osteogenic differentiation is critical for treatment of osteoporosis and MSC-based bone repair. In this study, we found that *GBP1* showed highest expression in all the GBPs in hBM-MSCs, and it was downregulated during osteogenic differentiation. While siRNA-mediated depletion of *GBP1* promoted osteogenic differentiation, ectopic overexpression of *GBP1* reduced osteogenic differentiation of hBM-MSCs. Furthermore, GBP1 is required for the expression of IDO, IL-6, and IL-8 induced by IFN-γ treatment, and knockdown of GBP1 rescued the inhibited osteogenic potential by IFN-γ treatment, at least in part.

To our knowledge, this is the first time that GBP1 has been found to inhibit osteogenesis of human MSCs. GBPs were initially identified as IFN-γ-responsive genes that mediate the antibacterial and antiviral activities of IFN-γ. In mice, mGBPs accumulate around pathogen-containing vacuoles (PV) and are important in restricting intracellular pathogens and immune activation, such as *Toxoplasma*^26^. However, recent study has also reported that hGBP1 restricted *Toxoplasma gondii*, but did not localize to pathogen vacuoles, revealing different mechanisms between hGBPs and mGBPs^27^. Furthermore, compared to other hGBPs, the crystal structure of human GBP-1 has revealed its different biochemical properties^16,28^. In this study, we also different expression patterns of hGBPs in hBM-MSCs exposed to osteogenic stimulus. For instance, GBP1 was dramatically downregulation, but GBP2 was upregulated during osteogenic differentiation of hBM-MSCs. More efforts are still needed to investigate whether the other GBPs play a role in regulation of lineage specification of MSCs.

Our results also revealed that GBP1 is required for IFN-γ signaling in hBM-MSCs. Previous studies have highlighted the role of GBPs in inflammasome activation and innate immune response^29,30^. And deletion of *Gbp1*, *Gbp2*, *Gbp3*, and *Gbp5* in macrophages often resulted in impaired immune response, respectively, indicating an important role of GBPs in inflammatory signaling^29,31,32^. In addition, several studies have shown that GBP1 can also mediate IFN responses that are not directly associated with host defense against pathogens, such as inhibition of matrix metalloproteinase expression^33^, inhibition of proliferation in endothelial cells^34^. Consistently, we found knockdown of *GBP1* significantly suppressed expression of *IDO*, *IL-6* and *IL-8* induced by treatment with IFN-γ in this study. However, knockdown of GBP1 did not fully rescue the inhibited osteogenesis of hBM-MSCs by IFN-γ treatment. It may be because of the redundancy among the GBPs, since all GBP-1 through -5 can be induced by IFN-γ treatment in hMSCs^20^. Finally, one of the limitations of this study is that how GBP1 regulate IFN-γ signaling in hBM-MSCs remain largely unknown. Future studies may focus on the mechanisms by which GBPs mediate IFN-γ signaling.

In conclusion, we have found GBP1 was downregulated during osteogenic differentiation of hBM-MSCs. While knockdown of *GBP1* promoted osteogenesis, overexpression of *GBP1* suppressed osteogenesis of hBM-MSCs. Furthermore, we found GBP1 is required for IFN-γ signaling, and depletion of *GBP1* rescued the inhibited osteogenesis induced by IFN-γ treatment, at least in part. Together with previous study, high expression of *GBP1* in monocytes and MSCs may be associated with the risk to osteoporosis, and inhibition of GBP1 expression may prevent development of osteoporosis and facilitate MSC-based bone regeneration.

## Electronic supplementary material

Supplementary Information
