# Supplementary material for: Guanylate Binding Protein 1 Inhibits Osteogenic Differentiation of Human Mesenchymal Stromal Cells Derived from Bone Marrow
Source: Sci Rep. 2018 Jan 18;8:1048. doi: 10.1038/s41598-018-19401-2 (PMC5773562; doi:10.1038/s41598-018-19401-2)
Supplement: Supplementary file 1 — Supplementary Information [file 41598_2018_19401_MOESM1_ESM.pdf]

Guanylate Binding Protein 1 Inhibits Osteogenic Differentiation of Human Mesenchymal Stromal Cells Derived from Bone Marrow

Shi Bai[1, 2, 3], Zhixiang Mu[1, 2, 3], Yuanding Huang[1, 2, 3], Ping Ji[1, 2, 3, *]

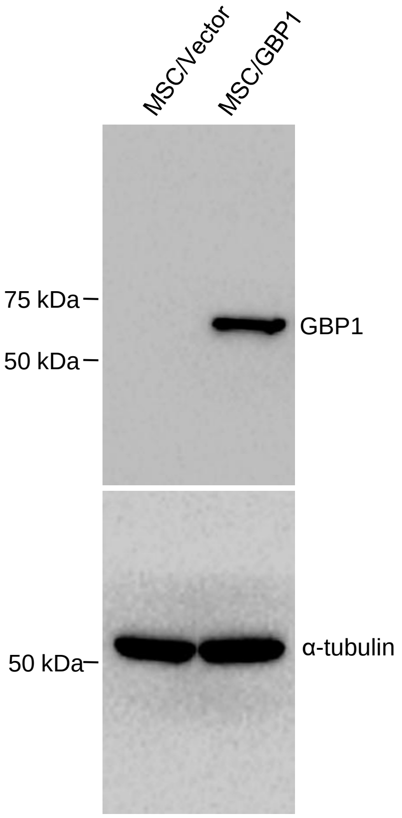

Figure S1. Original images of western blots displayed in Figure 3B.

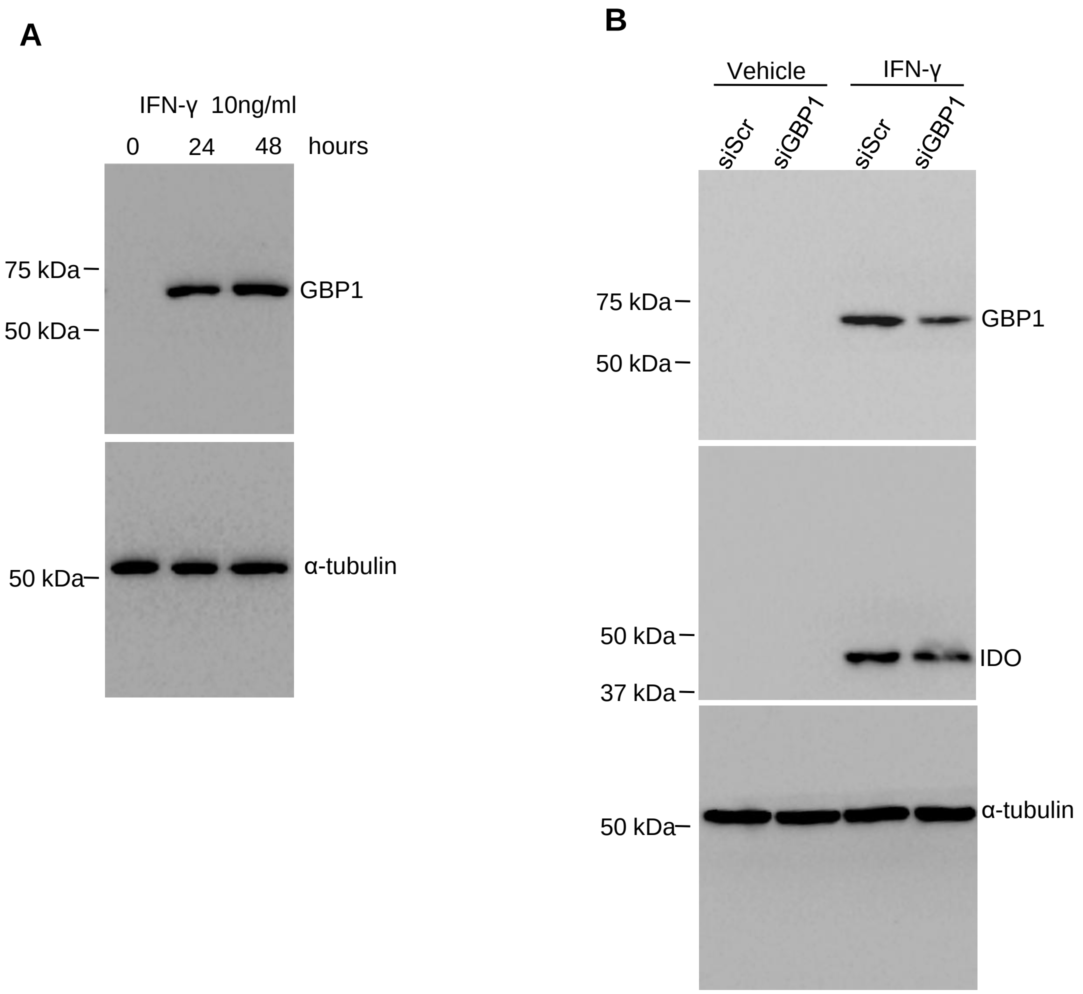

Figure S2. Original images of western blots displayed in Figure 4B (A) and Figure 4D (B).
